# CDAPubMed: a browser extension to retrieve EHR-based biomedical literature

**DOI:** 10.1186/1472-6947-12-29

**Published:** 2012-04-05

**Authors:** David Perez-Rey, Ana Jimenez-Castellanos, Miguel Garcia-Remesal, Jose Crespo, Victor Maojo

**Affiliations:** 1 Biomedical Informatics Group, Facultad de Informática, Departamento de Inteligencia Artificial, Universidad Politécnica de Madrid, Campus de Montegancedo, s/n, 28660 Madrid, Spain

## Abstract

**Background:**

Over the last few decades, the ever-increasing output of scientific publications has led to new challenges to keep up to date with the literature. In the biomedical area, this growth has introduced new requirements for professionals, e.g., physicians, who have to locate the exact papers that they need for their clinical and research work amongst a huge number of publications. Against this backdrop, novel information retrieval methods are even more necessary. While web search engines are widespread in many areas, facilitating access to all kinds of information, additional tools are required to automatically link information retrieved from these engines to specific biomedical applications. In the case of clinical environments, this also means considering aspects such as patient data security and confidentiality or structured contents, e.g., electronic health records (EHRs). In this scenario, we have developed a new tool to facilitate query building to retrieve scientific literature related to EHRs.

**Results:**

We have developed CDAPubMed, an open-source web browser extension to integrate EHR features in biomedical literature retrieval approaches. Clinical users can use CDAPubMed to: (i) load patient clinical documents, i.e., EHRs based on the Health Level 7-Clinical Document Architecture Standard (HL7-CDA), (ii) identify relevant terms for scientific literature search in these documents, i.e., Medical Subject Headings (MeSH), automatically driven by the CDAPubMed configuration, which advanced users can optimize to adapt to each specific situation, and (iii) generate and launch literature search queries to a major search engine, i.e., PubMed, to retrieve citations related to the EHR under examination.

**Conclusions:**

CDAPubMed is a platform-independent tool designed to facilitate literature searching using keywords contained in specific EHRs. CDAPubMed is visually integrated, as an extension of a widespread web browser, within the standard PubMed interface. It has been tested on a public dataset of HL7-CDA documents, returning significantly fewer citations since queries are focused on characteristics identified within the EHR. For instance, compared with more than 200,000 citations retrieved by *breast neoplasm*, fewer than ten citations were retrieved when ten patient features were added using CDAPubMed. This is an open source tool that can be freely used for non-profit purposes and integrated with other existing systems.

## Background

More than 700,000 biomedical articles were published in 2009 and indexed in MEDLINE [1]. At this rate, an internist like other medical specialists, in fact would have to read at least 20 scientific papers every day to keep up-to-date with this overwhelming number of yearly citations [2]. To address this information explosion, different kinds of search engines, such as PubMed [3] or HubMed [4], for instance, supplement biomedical literature databases. These engines provide instant access to the biomedical literature. However, the large volume of available citations often leads to the retrieval of too many results. PubMed, for example, retrieves citations ordered by date, but this criterion is rarely the main attribute used to measure the relevance of a biomedical publication. In practice, users need to perform further filtering and query modifications to retrieve the results that best suit their personal needs which might not be listed on the first page of results.

Looking back to the early years of the pioneering information retrieval systems, the number of records was much more limited. Furthermore, exact word-matching was usually enough to find the specific document that a user was searching for [5]. Nowadays, professionals require more advanced document indexation architectures and advanced natural language processing (NLP) techniques to handle the current information growth on the Web. To address this new situation, traditional information retrieval systems have evolved into modern search engines that can work in highly dynamic and large environments, e.g. Google [6], Yahoo [7] or Bing [8].

The above search engines were initially based on simple keyword-based queries. However, such queries were not efficient enough given the increasing complexity of the structures and contents of the current Web. Thus, user-related data, i.e., user contexts, such as physical location or query and web history, for instance, had to be built into commercial search engines since relevance measures are different for each user [9]. Regarding search engines for biomedical literature, recent efforts focused on improving citation retrieval from MEDLINE. For instance, systems such as HubMed [4], highlights articles that contain the search terms within the title or abstract, or Relemed [10] which uses sentence-level concurrence as a relevance metric for multi-word queries, askMEDLINE [11] allows free-text, natural language queries or GoPubMed [12] classify citations using Gene Ontology terms.

In other areas, the inclusion of user context information has improved the performance of search engines [13,14]. For instance, IntelliZap [15] addresses textual contexts. The input for this search engine tool is a set of keywords and a text based on natural language, whereas the output is a number of results, also related to the textual context. Other information retrieval systems can personalize their results according to user history [16,17], ontological knowledge [18,19] or user profiles [20], e.g., to perform different result rankings depending on user profile clusters [21].

In clinical practice, it is worthwhile examining a similar approach, i.e., considering user context information for retrieval purposes. For clinical applications, we might use information available within hospital information systems (HIS) and electronic health records (EHR) [22], for instance. In this regard, projects such as MorphoSaurus [23] and others [24] focused on concept-based and semantic approaches to improve medical information search and retrieval. InfoButtons [25] have been widely used to provide links to online resources from EHRs with MEDLINE among these resources [26]. However, there is still a lack of tools performing complex queries to retrieve biomedical literature. The process of query building, incrementally including terms for advanced filtering, is usually performed manually. We developed CDAPubMed to address this issue, aiming to provide a tool to semi-automatically build such complex queries.

The objective of CDAPubMed is to use the contents of EHRs to provide additional information for improving and personalizing biomedical literature searches. For instance, let us assume that there is a clinician searching biomedical citations for a given disease in a particular patient. The traditional process would be to access PubMed and search all the relevant citations by typing the specific keywords that the clinician considers most relevant for this case. After an initial usually generic search, he or she needs to reduce the scope of the search by using more specific terms related to a particular patient, until he or she finds the most relevant citations. This is usually a multi-step process and can, in fact, be tedious and time-consuming. In contrast, we suggest that the use of automated techniques can help this clinician to automatically find and filter these results, by linking citations with specific terms from the EHR that can be used to refine and improve query precision.

Following this approach, CDAPubMed is intended to help clinicians, researchers or other users to retrieve publications focused on their patients. When a user enters an EHR and a disease, the tool suggests and selects keywords within the EHR to filter the results. To accomplish this objective, the tool has to implement two main tasks: (i) automatic analysis of the EHR to identify relevant terms for literature retrieval, and (ii) generation of search engine queries to retrieve publications related to the EHR. Here we report our experience in developing CDAPubMed (available at http://porter.dia.fi.upm.es/cdapubmed/), and results showing its potential for EHR-based retrieval of biomedical literature.

## Implementation

### Architecture and technologies

**Figure 1:**
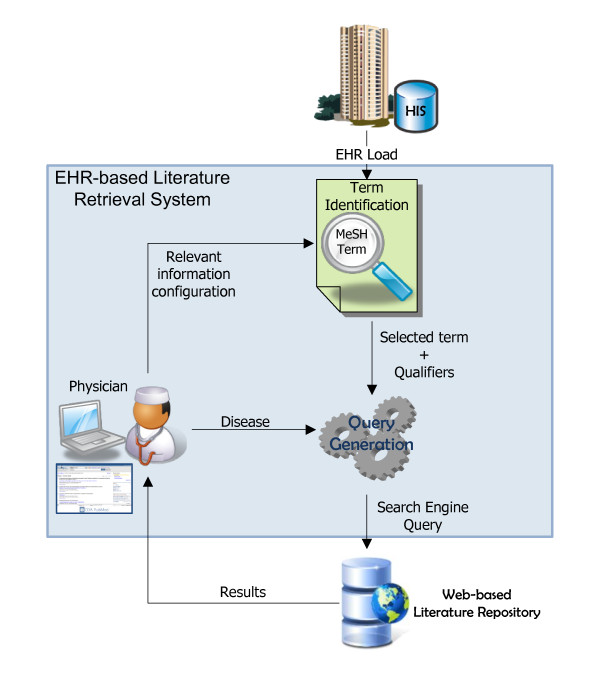
**An EHR-based literature retrieval system architecture**.

The EHR-based literature retrieval system proposed here and illustrated in Figure 1 requires two sources of information: (i) an EHR repository, and (ii) a biomedical literature search engine. The operation is initiated by selecting the EHR of a given patient from a local repository or HIS and a disease. The selected EHR is displayed to the user, highlighting relevant keywords for literature retrieval purposes. The user launches search queries by selecting the suggested terms. Following this approach, the system retrieves a number of citations, related to the given EHR.

Besides the above functionality requirements, the system architecture is also determined by security issues. Although security and anonymization protocols are available for sensitive data transfer, our approach is based on a client-side model to prevent patient data transmission to an external server. Note that the feasibility of this architecture depends on limiting the systems computational requirements to the possibilities of any device with a web browser.

CDAPubMed was developed as a Mozilla Firefox 3.6.x extension (or plug-in) resting on an architecture where all the information is processed on the client side. Firefox 3.6.x is the current version of the popular web browser maintained by Mozilla, which supports Java-based plugins. This being an open source project, Mozilla Firefox was selected because it is a widely accepted open source web browser. It also provides a hardware- and operating system- independent platform.

Regarding access to patient information, we implemented a wrapper for EHRs based on the Health Level 7-Clinical Document Architecture Standard (HL7-CDA). HL7-CDA is an eXtensible Markup Language (XML)-based format aiming to provide a structure for clinical information, but still including free text. A modular implementation has been carried out to facilitate the development of other XML-based interfaces within CDAPubMed's *term identification *module. In the current version, the *query generation *module generates PubMed queries, facilitating access to biomedical research publications. In addition, PubMed offers an application programming interface (API), i.e., Entrez Utilities [27], which is able to improve the performance of the system by retrieving specific information from PubMed, such as the number of citations instead of the full list of citations. Finally, the biomedical controlled vocabulary selected for CDAPubMed was the Medical Subject Headings (MeSH) thesaurus [28], developed by the National Library of Medicine (NLM) and used for article indexing in MEDLINE.

### Keyword Identification in HL7-CDA-Based EHRs

**Figure 2:**
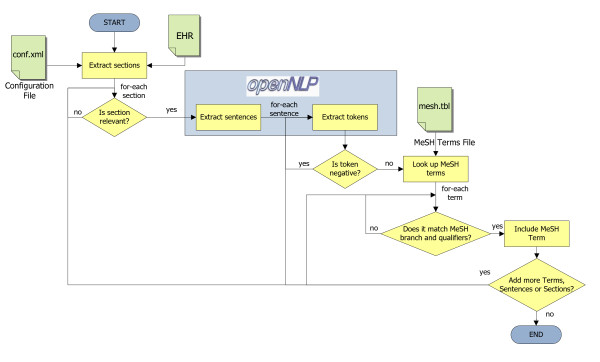
**The CDAPubMed algorithm for identifying relevant keywords in EHRs**.

The MeSH thesaurus is organized according to a tree-based structure, from general to specific concepts, containing in 2011, 177,000 Entry Terms and 26,142 Main Headings [28]. CDAPubMed's keywords identification algorithm requires the following information from the MeSH thesaurus: (i) the list of MeSH terms, (ii) a set of synonyms for each term, (iii) the MeSH tree branch or branches that include the MeSH term, and, to narrow down the MeSH term-related subject area, (iv) the corresponding set of allowable qualifiers [29], e.g., *Diabetes/epidemiology *will retrieve only epidemiological publications about *Diabetes*. Instead of using the original format provided by the NLM to store the required MeSH information, CDAPubMed employs a self-generated *hash table *[30] (labeled mesh.tbl in Figure 2) to improve the efficiency of keyword searching within the free text of HL7-CDA-based EHRs.

HL7-CDAs gather clinical data from different sources and are divided into sections, usually identified by LOINC or SNOMED codes [31]. CDAPubMed automatically identifies relevant MeSH terms, or synonyms, within each of these sections. However, not every MeSH term present in an EHR is necessarily relevant for citation retrieval. To meet the performance requirements of the PubMed API a default limit of 1,000 queries per minute CDAPubMed includes a configuration file (labeled conf.xml in Figure 2) with two mechanisms to avoid unnecessary keyword identifications. The first mechanism is the option of declaring an EHR section as relevant for literature retrieval purposes. Each section can be identified by a title, a set of synonym titles and/or LOINC/SNOMED codes. The identification process is confined to those sections declared as relevant only. EHRs sections such as *Appointment *or *Chief complaint *could be discarded for literature retrieval purposes. The second mechanism is to confine the identification process of an EHR section to a subset of MeSH terms, i.e., MeSH tree branches. For instance, the term *Penicillin *does not have the same implications when it is located in the *Allergies *section or when it is located in the section *Family Diseases*. Complete MeSH branches such as *Publication Characteristics (V)*, *Humanities (K) *or *Technology, Industry, Agriculture (J) *for instance, could also be discarded within the identification process if users consider that they are not relevant. CDAPubMed contains a default set of relevant sections and branch associations, those that obtained the best performance in the result section. Anyway, advanced users have the option of modifying this configuration through a graphical interface. The identification process, shown in Figure 2, is driven by such configuration.

For each relevant section of the EHR, CDAPubMed analyzes the content using an NLP package, i.e., OpenNLP [32], extracting sentences and words (or tokens) from the free text content. OpenNLP is an open source and machine learning-based toolkit, including a variety of java-based NLP tools, used for the processing of natural language text. At CDAPubMed, if a token, or consecutive tokens, is a MeSH term or synonym of the corresponding set of MeSH branches, this term is considered relevant for publication retrieval purposes, unless it is preceded by a negative expression, e.g. no *Pain*. Keywords identified by this algorithm are highlighted within the CDAPubMed interface (described in the CDAPubMed functionality section) and may trigger the PubMed query generation.

### Query generation

To generate a query through the CDAPubMed interface, the user may select one of the MeSH terms or synonyms highlighted in the EHR. For each selection, the matching keyword is added to the query, restricting the results to those related with the EHR. The relationship degree between a citation and an EHR implemented in CDAPubMed is calculated as follows:

$$Rel=\left|MeSH_{1}\left(pub\right)\cap \left(MeSH_{2}\left(ehr\right)\cup disease\right)\right|$$

A citation is related with an EHR by the intersection of MeSH terms indexing the publication, and the union of disease and relevant MeSH terms identified within the EHR.

The first iteration of CDAPubMed, i.e., the first time a MeSH term is selected after loading an EHR, generates the following query: (term_1_[mh]) AND (disease[mh]). The [mh] suffix specifies that the retrieved documents will be indexed by *term*_1 _in this case. Additional *n *keywords from the EHR can be integrated into the query in successive iterations: (term_1_[mh]) AND (term_2_[mh]) AND ...AND (term_*n*_[mh]) AND (disease[mh]). Note that keywords are linked with a conjunction connective (AND), instead of a disjunction connective (OR), to restrict the retrieved citations to the maximum relationship degree in each iteration. The *n^th ^*query in CDAPubMed retrieves citations related by *n + 1 *keywords to the EHR, or above, instead of citations related only by the disease.

To provide further restrictions within the same query level, qualifiers associated with MeSH terms and the respective EHR section can be selected. A qualifier is highlighted for selection within the interface, and the MeSH term is displayed in a different color if the qualifier is admissible for both the EHR section and the MeSH term. Allowable qualifiers for MeSH terms are taken from official MeSH information, whereas allowable qualifiers for sections are provided by the CDAPubMed configuration file. If a set of *m *qualifiers are selected for a set of *n *terms, the following query is generated: (term_1_/qualifier_1_) AND (term_1_/qualifier_2_) AND ...AND (term_*n*_/qualifier_*m*_) AND (disease). Citations for these queries are retrieved from PubMed or from a cache included within CDAPubMed. This cache stores a history of queries and the corresponding citations to improve system performance.

### CDAPubMed interface and functionality

**Figure 3:**
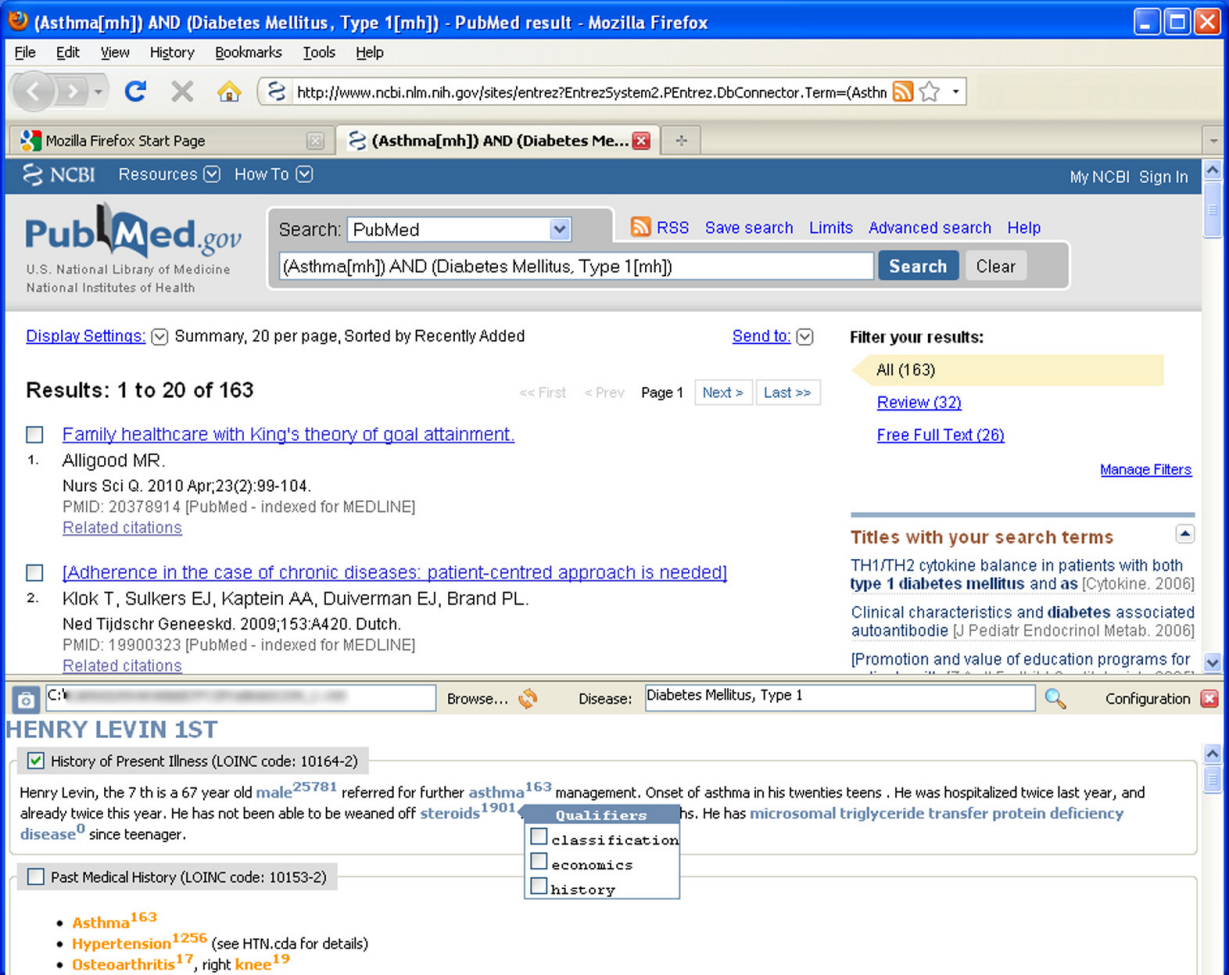
**CDAPubMed main interface**.

After a one-click installation of the CDAPubMed extension in Mozilla Firefox, a new window section at the bottom of the web browser can be activated, as shown in Figure 3. The rest of the browser window can be used for regular web browsing or PubMed result visualization. In the new area, the sequence of actions open to the user is: (i) load and validate a HL7-CDA file that will be displayed in the new window section, (ii) modify the default configuration of the MeSH identification process, (iii)enter a default disease from the C MeSH branch (Diseases), (iv) select one of the keywords within specific EHR sections to generate a PubMed query, and (v) restrict the resulting citations by selecting qualifiers for MeSH terms (if allowed). To iteratively retrieve more specific citations, the user may repeat action (iv) and select additional keywords from the EHR.

The screenshot illustrated in Figure 3 was the result of a query generated by CDAPubMed including the following keywords: *Diabetes Mellitus, Type 1 *(disease), *Asthma *and *Hypertension *(keywords selected from the EHR). In this case, CDAPubMed retrieved *6 *results related by at least *3 *relevant keywords with the EHR, instead of *53,137 *results retrieved by the *Diabetes Mellitus, Type 1 *general query.

The CDAPubMed interface highlights keywords that are considered relevant by the identification algorithm, including the potential number of retrieved publications displayed as a superscript next to the keyword. If there are qualifiers that can be applied to a MeSH term, this keyword is also highlighted (in a different color) and a menu, listing the allowable qualifiers, is displayed next to the respective term. When one or more qualifiers are selected for a MeSH term, the number of potential results is updated for this keyword. This number is refreshed for every keyword highlighted in the EHR, every time a query is made returning the number of citations that would be retrieved if the keyword is AND-concatenated to the query.

**Figure 4:**
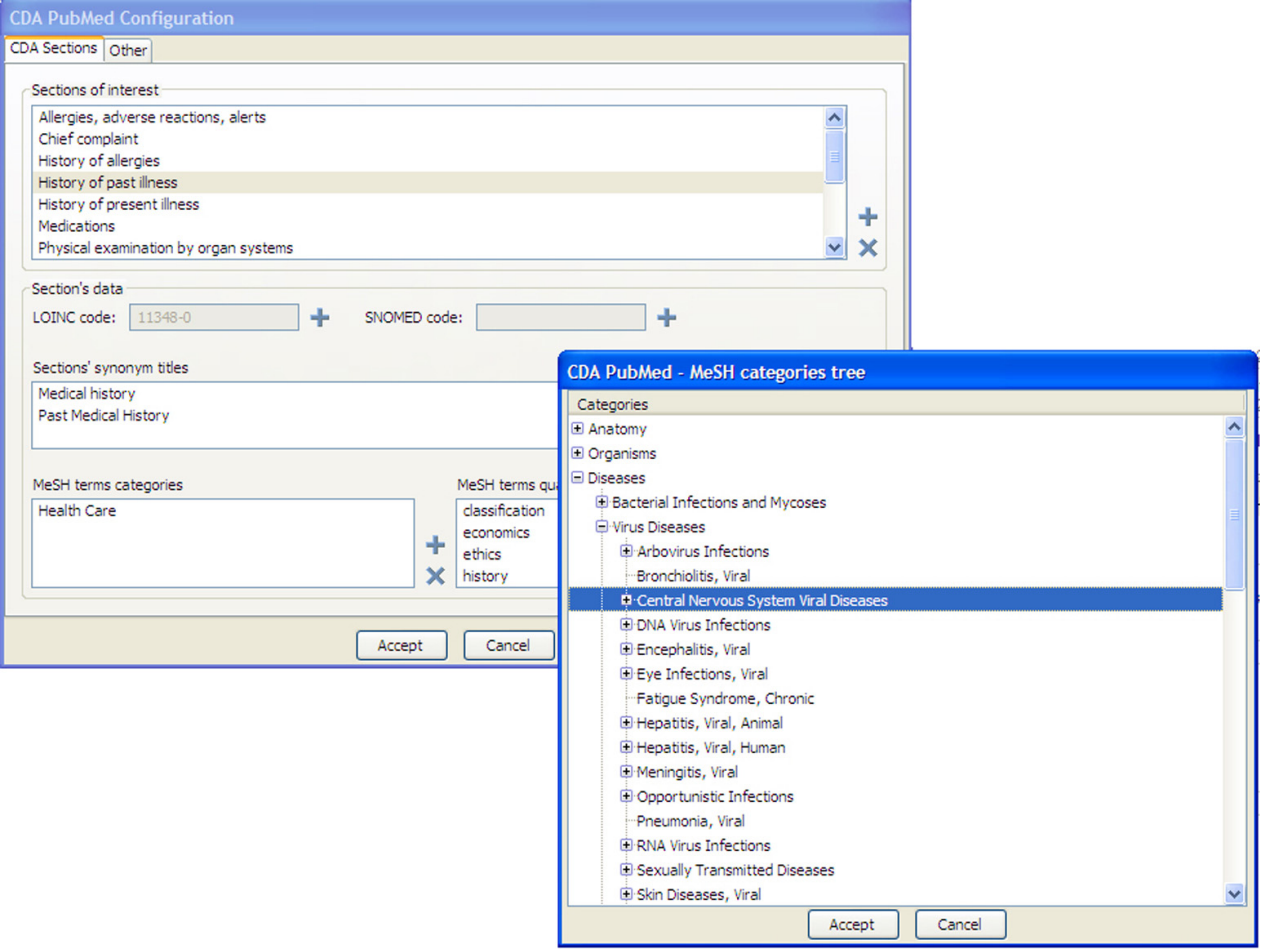
**CDAPubMed configuration tool**.

As mentioned in the previous section, the process of identifying and highlighting terms within the EHR can be optimized by setting some parameters of CDAPubMed. A graphical user interface (GUI) has been developed to modify the system configuration, as shown in Figure 4.

Users can use the CDAPubMed configuration tool to perform four main actions: (i) enter titles, synonyms or LOINC/SNOMED codes to identify new relevant sections for publication retrieval purposes, (ii) declare a set of MeSH term branches to search within a certain section., (iii) add a set of allowable qualifiers in a section, and, finally, (iv) save or load a configuration file to import/export a personal system configuration. A selection box available next to each EHR section in the general-purpose CDAPubMed interface is used to enter the title of a relevant section in the configuration. If no branches are associated to the MeSH term declaration, nine of the sixteen MeSH branches, A - G (Anatomy-Biological Sciences), N (Health Care) and Z (Geographic Locations) are associated by default, since they contain the most popular keywords for PubMed citation indexing. Finally, all qualifiers are used if no qualifiers are explicitly associated to a section. These actions are able to modify all the parameters that have an influence on CDAPubMed's keyword identification algorithm.

## Results

CDAPubMed has been tested on 17 HL7-CDA documents selected from a dataset of 74 HL7-CDA-based documents [33] and the 2011 MeSH version. The full dataset contains EHRs, laboratory reports, discharge summaries and urgent care summaries from different hospitals all over the world. We discarded clinical documents in languages other than English and containing mostly multimedia data. A clinician reviewed each EHR and identified every relevant term for biomedical publication retrieval. Table 1 shows the mean precision (P) and recall (R) of the relevant keyword identification process for different configurations of CDAPubMed in the dataset. If A-Z MeSH branches are included in the process almost every relevant keyword is detected, although 1/3 of the terms are not relevant. We have selected branches A-G within the default configuration, since they present more balanced results between precision and recall. Dictionary pruning also prevents unnecessary queries to the search engine, but advanced users may modify this configuration depending on their preferences. We have also applied a baseline method to identify every MeSH term occurrence within the dataset. Terms discarded due to negative tokens were included in this case, providing similar results to CDAPubMed identification method using every MeSH branch.

**Table 1:** Performance of the keyword identification process

MeSH branches	P	R
A-Z	97	68

A-G, N, Z	86	66

**A-G**	82	80

A-D, F	62	81

Baseline method	100	66

Mean Precision (P) and Recall (R) for 17 test EHRs (available at http://porter.dia.fi.upm.es/cdapubmed/download/) using four different groups of MeSH branches as dictionary (A: Anatomy, G: Biological Sciences, N: Health Care and Z: Geographic Locations)

**Figure 5:**
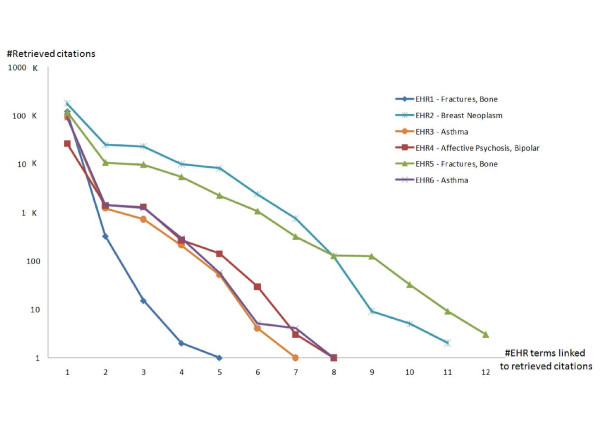
**Citation reduction specific queries to EHRs are generated in CDAPubMed**. The selected dataset is available at http://porter.dia.fi.upm.es/cdapubmed/download/. A logarithmic scale was applied to the vertical axis (number of retrieved citations).

After the keyword identification process, users may select keywords to be included in the query generation process, considering the number of publication that the new query would retrieve (a superscript number next to each keyword). Figure 5 illustrates the reduction of retrieved citations (performed on February 2011), compared to original queries, by focusing on six EHRs of the dataset in CDAPubMed. The disease used for each EHR was obtained from the respective *Present Illness *section. These results show that by using CDAPubMed, users can identify ten citations, i.e., a typical search engine first page of results, for an EHR and disease, mostly within four or five iterations. In cases with an extremely large number of MeSH terms within the EHR, i.e., over 200 keywords, up to ten iterations were necessary.

**Figure 6:**
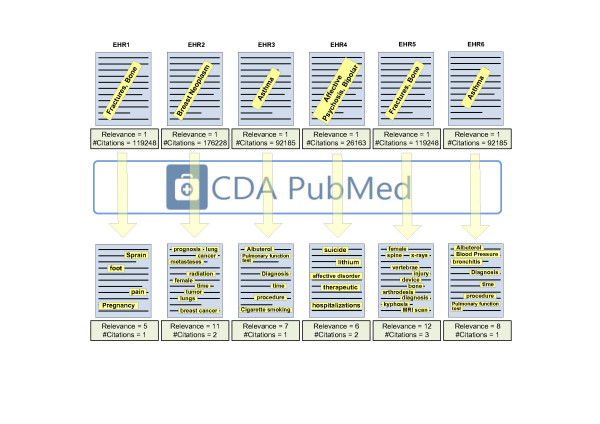
**Keywords used to return citations related by the maximum number of keywords with an EHR for a given disease**.

The results of initial queries are presented at the top of Figure 6 and the set of MeSH terms required to refine the original query are depicted below. Using CDAPubMed, citations are filtered by the context of clinical users, i.e., the EHRs of their patients. Unlike all citations retrieved by initial queries, EHR-based results are manageable by users, i.e. physicians.

A user satisfaction survey was performed to assess the usability of CDAPubMed. The three main tasks, required to use CDAPubMed, were evaluated: (i) Installation, (ii) Search and (iii) Configuration. Five external experts, a number suggested by [34], provided the feed-back presented on Table 2. Regarding the installation task, some experts commented on the requirement of a legacy version of Firefox. The 3.6.x version of Firefox was required since it is, at the time of writing this paper, the last one supporting Java-based plugins, and CDAPubMed requires a Java-based NLP library, i.e OpenNLP. The literature search task was highly rated, highlighting the intuitive use of CDAPubMed on the default configuration. While the configuration usability was rated slightly lower, since it required advanced knowledge of the tool, HL7-based EHRs and MeSH terminologies. It should be noted that the configuration module is optional and intended for advanced users to optimize CDAPubMed performance.

**Table 2:** Overall impression from external experts of each CDAPubMed task (1 = Very negative, 5 = Very positive)

CDAPubMed task	Expert 1	Expert 2	Expert 3	Expert 4	Expert 5
Installation	5	2	5	4	4

Search	4	4	5	4	4

Configuration	4	2	5	4	3

Online survey and full results are available at http://porter.dia.fi.upm.es/cdapubmed/user-satisfaction-survey/

## Discussion

In recent years, different extensions has been designed for web browsers to provide a mechanism of user and context-based customization for many different computing applications, such as PubMed-EX [35] to mark-up gene/disease information in PubMed results. They include new features for Internet navigation, but also for adapting the behavior of web-based systems, such as web search engines, to the user preferences. Web browser customization has not yet been widely applied to biomedicine.

Some recent developments have focused on improving the results of biomedical search engines, such as PubMed, by implementing relevance-based methods [36]. But there is still a shortage of methods to personalize these results by linking them to specific user contexts.

CDAPubMed was developed as one such web browser extension, providing an intuitive and configurable tool to enhance PubMed results based on patient features. These features are extracted in this case from HL7-CDA EHRs, but other XML-based standards for EHRs, such as new versions of the HL7-CDA, will provide new possibilities to identify relevant retrieval terms within more structured documents. However, optimal default configurations should be always provided and new challenges will arise to manage the configuration of such tools.

In CDAPubMed, users can select relevant keywords to semi-automatically build queries, discovering publications related to EHR. The number of publications, that each keyword would retrieve if added to the query, is displayed next to each relevant term to facilitate keyword selection. MeSH major topics and existing machine learning-based methods to automatically extract categories [37], or the most relevant MeSH terms for biomedical literature indexing [38,39], could also be applied in future implementations of EHRs to prioritize relevant terms within CDAPubMed.

Compared to similar projects such as InfoButtons [25] or the Medline Button [26], CDAPubMed is the only tool available as a browser extension and using PubMed as the search interface. But CDAPubMed also provides a different approach for EHR-based literature retrieval. Instead of providing links to external resources within the EHR, i.e. InfoButtons, or providing a list of possible questions related to the EHR, i.e. the Medline Button, CDAPubMed allows to build complex queries on the most used MEDLINE search interface. In addition, CDAPubMed queries are constructed by selecting MeSH terms and qualifiers from EHRs, providing additional information to the user related to the potential number of retrieved publications for each term.

## Conclusions

The objective of this research was to provide a tool that can improve the literature retrieval performed by clinical professionals. CDAPubMed facilitates the identification of citations related to patient characteristics by including EHR features within the PubMed query, a traditionally manual process. The technologies used in this work ensured independence from hardware platforms and operating systems. This will improve the adoption of CDAPubMed in environments such as education, research or clinical practice.

Future applications of CDAPubMed would benefit from extending the current version with (i) new wrappers for additional EHR formats such as Continuity of Care Record (CCR), CEN-13606 or openEHR, (ii) new modules for query generation to fetch publications from other search engines, (iii) a new communication layer to load EHRs from HIS databases instead of a local file, or (iv) a method to automatically find and launch the query by retrieving publications related to the EHR with the maximum number of relevant available terms although such method may also obtain too focused citations. Some of these improvements, such as the last one, should address the performance limitations of search engine APIs specific for literature. Other improvements, such as incorporating information from other sources, i.e., HIS, new EHR formats or new search engines, will call for the management of different information standards. As CDAPubMed is an open source tool and has a modular architecture, future integrations, code reuse and contributions from the biomedical informatics community will be feasible.

## Availability and requirements

Project name: CDAPubMed

Project home page: porter.dia.fi.upm.es/cdapubmed

Operating systems: Platform independent

Programming language: Java, Javascript and XUL

Other requirements: Mozilla Firefox Browser with Java Plugin

License: Open Source

Any restrictions to use by non-academics: Incorporation into commercial products restricted

## Abbreviations

EHR: Electronic health record; HL7-CDA: Health level 7-clinical document architecture; MeSH: Medical subject headings; NLP: Natural language processing; HIS: Hospital information system; XML: eXtensible markup language; API: Application programming interface; LOINC: Logical observation identifiers names and codes; SNOMED: Systematized nomenclature of medicine; GUI: Graphical user interface.

## Competing interests

The authors declare that they have no competing interests.

## Authors' contributions

DPR conceived the tool and evaluation. DPR and AJ designed and participated in the development of the tool, ran the evaluation and drafted the manuscript. MGR participated in the design of the tool and manuscript preparation. JC provided feedback about the CDAPubMed functionality and manuscript. VM participated in the evaluation, contributed to manuscript preparation and approved the final version for publication. All authors read and approved the final manuscript.

## Pre-publication history

The pre-publication history for this paper can be accessed here:

http://www.biomedcentral.com/1472-6947/12/29/prepub
